# KunTai capsules combined with Femoston in premature ovarian insufficiency: a randomized controlled trial of the effects on bone mineral density

**DOI:** 10.3389/fendo.2026.1746993

**Published:** 2026-03-26

**Authors:** Zijie Fu, Xueping Liu, Qingya Ma, Tongyao Geng, Ying Zheng, Yujing Wang, Yahui Bian, Wei Guo, Xiaodong Li

**Affiliations:** Department of Gynecology, The First Hospital of Hebei Medical University, Shijiazhuang, China

**Keywords:** bone mineral density, bone turnover markers, hormone levels, modified K score, premature ovarian insufficiency

## Abstract

**Purpose:**

This study aims to evaluate the clinical efficacy of KunTai capsules combined with Femoston on bone mineral density (BMD) in women with premature ovarian insufficiency (POI).

**Methods:**

This prospective, randomized, controlled clinical trial was conducted at the First and Second Hospitals of Hebei Medical University from September 2018 to July 2022. Participants were randomly assigned in a 1:1 ratio to the control (Femoston alone) or the observation group (Femoston + KunTai capsules). Treatment duration was 12 months. The primary endpoint was the change in lumbar spine BMD at 12 months. Secondary endpoints included bone turnover markers (P1NP, β-CTX), hormone levels (E_2_, FSH, LH), and modified Kupperman scores.

**Results:**

A total of 104 women were randomized, and 73 completed the 12-month follow-up. After 12 months of treatment, the observation group demonstrated significant increases in total lumbar spine and L1-L4 BMD compared with baseline (*P* < 0.05). In addition, BMD changes at total lumbar spine, L2, and L3 were significantly greater compared to the control group (*P* < 0.05). In terms of bone turnover markers (P1NP and β-CTX), no significant differences were observed either between the two groups or compared with baseline after treatment (*P* > 0.05). In the control group, E_2_ levels were significantly increased at 3, 6, and 12 months compared with baseline (*P* < 0.05), while FSH levels were significantly decreased at 3 and 6 months (*P* < 0.05) but showed no significant difference at 12 months. In the observation group, compared with baseline, E_2_ levels were significantly increased, whereas FSH and LH levels were significantly decreased at 3, 6, and 12 months (*P* < 0.05). After 12 months of treatment, improvements in modified Kupperman scores were significantly greater in the observation group than in the control group (*P* < 0.05).

**Conclusion:**

The combination of KunTai capsules and Femoston was associated with a greater increase in lumbar spine BMD at 12 months compared with Femoston monotherapy in women with POI. Improvements in menopausal symptoms were also observed; however, no significant between-group differences were found in bone turnover markers or hormone levels.

**Clinical Trial Registration:**

https://www.chictr.org.cn/showproj.html?proj=26556, identifier (ChiCTR1800017774).

## 1 Introduction

Premature ovarian insufficiency (POI) is a condition in which women develop amenorrhea or hypomenorrhea before the age of 40, accompanied by elevated gonadotropin levels and decreased estradiol (1). A recent meta-analysis published in 2023 revealed that the global overall prevalence of POI among women was as high as 3.5% (2). Women with POI may present with menstrual changes, menopausal symptoms, infertility, and even increased long-term risk of cardiovascular disease and osteoporosis due to low estrogen (3, 4). Compared with women who undergo menopause at the usual age, those with POI have a significantly lower bone mineral density (BMD), a higher risk of osteoporosis and a 1.36-fold greater risk of fracture (5–7). These findings underscore the importance of maintaining bone health in women with POI.

Current guidelines consistently recommend that hormone replacement therapy (HRT) should be initiated in women with POI and continued at least until the average age of natural menopause (8). Among estrogen replacement options, 17 β-estradiol is preferred (9). Prior studies have demonstrated that the combination of estradiol with dydrogesterone provides effective relief of menopausal symptoms and prevention of osteoporosis, while maintaining a favorable safety profile (10). However, it has been reported that standard-dose HRT appears to be inadequate to reduce bone loss in young women with POI (11). Additionally, some patients are reluctant to use HRT due to contraindications or concerns about possible adverse events such as thromboembolic events and breast cancer. Therefore, there is a pressing need to explore and optimize treatment strategies in women with POI.

Traditional Chinese medicine (TCM) has a long history of use in China to improve female reproductive function. KunTai Capsules, a TCM preparation for the treatment of ovarian function decline, have been shown to improve sex hormone levels and effectively relieve menopausal symptom (12). In addition, KunTai Capsules combined with Raloxifene can significantly reduce the bone turnover rate in postmenopausal women with osteoporosis and improve BMD in the hip and lumbar spine (13).

This study aimed to evaluate the effects of KunTai capsules combined with Femoston on bone mineral density in women with POI and to assess changes in hormone levels and bone turnover markers following treatment.

## 2 Materials and methods

This prospective, randomized, controlled clinical trial recruited women with POI between September 2018 and July 2022 at the First Hospital of Hebei Medical University and the Second Hospital of Hebei Medical University in China. The study protocol was approved by the ethics committees of both hospitals (Approval No. 2018-C010 and 20220588). All participants provided written informed consent prior to enrollment. The study was registered at Chinese Clinical Trial Registry (Registration No.ChiCTR1800017774, Registered 13 August 2018, https://www.chictr.org.cn/showproj.html?proj=26556). No participants were recruited prior to trial registration.

### 2.1 Participants

Women aged 18–40 diagnosed with POI were eligible to participate. POI was defined according to the ESHRE guideline as oligo or amenorrhea for at least 4 months, accompanied by elevated FSH levels >25 IU/L on two separate occasions >4 weeks apart (1). Exclusion criteria included: any absolute contraindications to hormone therapy; taking any medication known to affect BMD or with a condition affecting BMD; severe diseases of other systems.

### 2.2 Randomization

After confirmation of eligibility and written informed consent, participants were randomized using a computer-generated random sequence to be in either the control group or the observation group. Allocation concealment was ensured using sequentially numbered, sealed, opaque envelopes. Due to the nature of the intervention, this was an open-label study.

### 2.3 Intervention

The control group (Femoston) was treated with Femoston, consisting of continuous 17 β-estradiol (2 mg/day) combined with sequential dydrogesterone (10 mg/day for 14 days per cycle, Abbott Biologicals B.V.). The observation group (Femoston+HYKT) received the same Femoston in combination with oral Kuntai Capsules (4 capsules per dose, 3 times daily, Guiyang Xintian Pharmaceutical Co., Ltd., China). The selected dosage was based on the manufacturer’s recommended instructions and is consistent with routine clinical practice. The total treatment duration was 12 months.

### 2.4 Outcome measures and procedures

Participants were followed at the First and the Second Hospitals of Hebei Medical University and were assessed at baseline and at 3, 6 and 12 months of treatment. Compliance was monitored by requesting participants to return medication packets and documenting any missed or unused doses. The primary endpoint was the change in lumbar spine BMD from baseline after 12 months of treatment in the two groups. Given the relatively slow nature of bone remodeling, lumbar spine BMD was assessed at baseline and after 12 months of treatment only. Serum hormone levels (E_2_, FSH, and LH), bone turnover markers (procollagen type I N-terminal propeptide (P1NP) and C-terminal crosslinked telopeptide (β-CTX)), and the modified Kupperman index were also measured at baseline, 3, 6, and 12 months. All BMD measurements were performed using dual-energy X-ray absorptiometry scan. Fasting blood samples collected at each visit were used to measure P1NP and β-CTX via the Roche Elecsys system, and sex hormones were quantified using electrochemiluminescence immunoassay (ECLIA) on a Beckman automated analyzer.

### 2.5 Sample size

The sample size was calculated based on the expected difference in lumbar spine BMD change between groups. According to previous studies, a mean difference of 0.02 g/cm² with a standard deviation of 0.029 g/cm² was assumed. With a two-sided α of 0.05 and a power (1−β) of 80%, and assuming a 1:1 allocation ratio, 34 participants were required in each group. Considering an anticipated dropout rate of 20%, a minimum of 86 participants was needed. To ensure an adequate number of study participants, a total of 104 participants were planned for randomization.

### 2.6 Statistical analysis

All statistical analyses were performed using SAS 9.4 software. *P* < 0.05 was considered statistically significant. Efficacy analyses were based on the Full Analysis Set (FAS), which included all randomized participants who received at least one dose of study medication and had at least one post-baseline assessment. The primary endpoint, the change in lumbar spine BMD from baseline to 12 months, was analyzed using all available data within the FAS.

Baseline characteristics were compared between groups using an independent samples t-test for normally distributed variables or the Mann-Whitney U test for non-normally distributed variables. Within-group comparisons between post-treatment and baseline were conducted using a paired t-test or the Wilcoxon signed-rank test. Between-group comparisons of the primary and secondary endpoints were performed using an independent samples t-test or a Mann-Whitney U test based on data distribution.

## 3 Results

### 3.1 Enrolment

**Figure 1:**
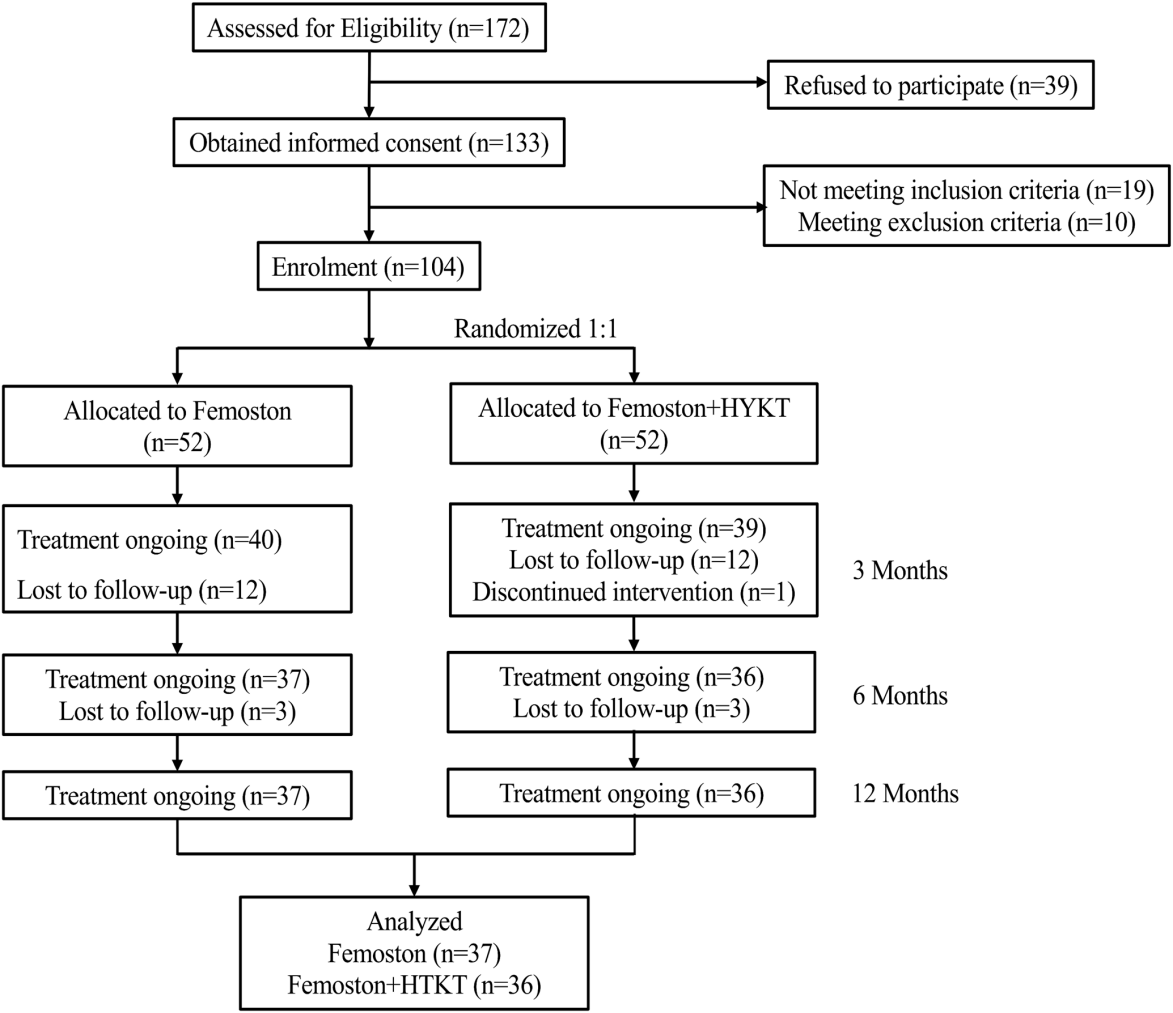
Flow diagram of the study.

The study flowchart of the participants is shown in **Figure 1**. From September 2018 to July 2022, a total of 172 women were assessed for eligibility. Of these, 39 declined participation and 29 did not meet the inclusion or exclusion criteria, leaving 104 participants who were randomized in a 1:1 ratio to the control group (n=52) and the observation group (n=52). During the 12-month follow-up period, 15 participants in each group were lost to follow-up. One participant in the observation group discontinued the intervention due to nonadherence and was not included in the final analysis. Ultimately, 73 patients (37 in the control group and 36 in the observation group) completed the 12-month follow-up and had available BMD data. The overall dropout rate at 12 months was 29.8%.

### 3.2 Baseline data

The baseline characteristics of the study participants are shown in **Table 1**. The mean age was 26.67 ± 7.02 years in the Femoston group and 24.97 ± 7.12 years in the Femoston+HYKT group (*P* = 0.2063). And there were no significant differences in baseline levels of sex hormones, including E_2_ (*P* = 0.2434), FSH (*P* = 0.1467), and LH (*P* = 0.6311). No significant differences were observed between the two groups in other baseline measures, including P1NP, β-CTX, ALP and the K score (*P* > 0.05).

**Table 1:** Baseline characteristics for treatment groups.

Characteristics	Femoston (n=40)	Femoston+HYKT (n=39)	*P*
Age (years)	26.67 ± 7.02	24.97 ± 7.12	0.2063
E_2_ (pg/mL)	25.00 (13.50,39.50)	25.00 (13.50,39.50)	0.2434
FSH (IU/L)	76.84 (52.19, 98.29)	80.57 (52.69, 131.79)	0.1467
LH (IU/L)	29.65 (19.50,45.19)	29.65 (19.50,45.19)	0.6311
BMD LS (g/cm^2^)	0.83 ± 0.10	0.78 ± 0.11	0.0072
BMD FN (g/cm^2^)	0.65 ± 0.14	0.64 ± 0.08	0.2109
BMD LH (g/cm^2^)	0.82 ± 0.10	0.78 ± 0.08	0.0551
P1NP (mmol/L)	82.48 (59.45,132.50)	82.48 (59.45,132.50)	0.5250
β-CTX (ng/mL)	0.33 (0.25, 0.45)	0.43 (0.24, 0.59)	0.2309
ALP	84.00 (66.40,106.20)	84.00 (66.40,106.20)	0.9038
K score	8.00 (2.25, 13.00)	8.00 (3.50, 11.25)	0.9599

Data are mean (SD) or median (IQR). HYKT, KunTai Capsules; BMD LS, bone mineral density of the lumbar spine; BMD FN, bone density measurement of the femoral neck; BMD LH, bone density measurement of the left hip; P1NP, procollagen type 1 N-terminal propeptide; CTX, C-telopeptide of type 1 collagen.

As for BMD, the Femoston+HYKT group had a significantly lower mean lumbar spine BMD (BMD LS) (0.78 ± 0.11 g/cm^2^) compared to the Femoston group (0.83 ± 0.10 g/cm^2^) (*P* = 0.0072). While the baseline BMD of the femoral neck (BMD FN) and the left hip (BMD TH) showed no significant differences between the two groups.

### 3.3 Primary outcome-lumbar spine bone mineral density

The primary outcome of this study was to evaluate the change in BMD LS after 12 months of treatment.

After 12 months, the total BMD LS in both groups showed significant improvements from baseline (**Table 2**). In the Femoston group, the total BMD LS increased from 0.83 ± 0.10 g/cm^2^ to 0.85 ± 0.10 g/cm^2^. In the Femoston+HYKT group, it increased from a baseline of 0.78 ± 0.11 g/cm^2^ to 0.81 ± 0.10 g/cm^2^. Significant increases were also observed at each individual lumbar vertebral level (L1-L4) in the Femoston+HYKT group (*P* < 0.05).

**Table 2:** Comparison of lumbar spine BMD before and after 12 months of treatment in each groups.

Group	Total		L1		L2		L3		L4	
	Before	After	Before	After	Before	After	Before	After	Before	After
Femoston	0.83 ± 0.10	0.85 ± 0.10^*^	0.77 ± 0.11	0.80 ± 0.11^*^	0.82 ± 0.11	0.85 ± 0.11^*^	0.86 ± 0.10	0.88 ± 0.10^*^	0.84 ± 0.09	0.86 ± 0.09
Femoston+HYKT	0.78 ± 0.11	0.81 ± 0.10^*^	0.72 ± 0.12	0.75 ± 0.11^*^	0.78 ± 0.12	0.81 ± 0.11^*^	0.81 ± 0.11	0.84 ± 0.11^*^	0.81 ± 0.11	0.83 ± 0.10^*^

*P < 0.05 vs. baseline within the group.

The between-group comparison of changes in BMD LS after 12 months of treatment showed a significant difference in efficacy. As shown in **Table 3**, the mean increase in total BMD LS in the Femoston+HYKT group (0.05 ± 0.04 g/cm^2^) was significantly greater than the Femoston group (0.02 ± 0.03 g/cm^2^) (*P* = 0.016). Further analysis of individual vertebral showed that the BMD increases at L2 (*P* = 0.017) and L3 (*P* = 0.038) were significantly greater in the Femoston+HYKT group compared to the Femoston group.

**Table 3:** Between-group comparison of changes in lumbar spine bone mineral density after 12 months of treatment.

Group	Total	L1	L2	L3	L4
Femoston	0.02 ± 0.03	0.03 ± 0.06	0.02 ± 0.04	0.02 ± 0.04	0.02 ± 0.04
Femoston+HYKT	0.05 ± 0.04	0.06 ± 0.06	0.05 ± 0.04	0.04 ± 0.04	0.04 ± 0.04
*P*	0.016	0.115	0.017	0.038	0.059

These findings suggest that treatment with KunTai capsules combined with Femoston is more effective at increasing BMD LS than Femoston alone in women with POI.

### 3.4 Secondary outcomes

#### 3.4.1 Modified Kupperman index (K score)

The change in the modified K score from baseline was assessed at 3, 6, and 12 months to evaluate the improvement of menopausal symptoms. As shown in **Table 4**, both groups exhibited a reduction in K scores over the 12-month treatment period. At the 3- and 6-month follow-ups, the decrease in K scores was greater in the Femoston+HYKT group compared with the Femoston group, but the differences were not statistically significant (*P* = 0.808 and *P* = 0.164, respectively). At the 12-month follow-up, however, the reduction in the K score was significantly greater in the Femoston+HYKT group than in the Femoston group (−8.25 ± 2.87 vs. −0.75 ± 0.96, *P* = 0.029), indicating that the combination therapy was more effective at alleviating menopausal symptoms.

**Table 4:** Between-group comparison of change in modified K score at 3, 6, and 12 months.

Group	3 Months	6 Months	12 Months
Femoston	-2.78 ± 3.31	-1.25 ± 2.50	-0.75 ± 0.96
Femoston+HYKT	-2.83 ± 5.95	-6.00 ± 8.98	-8.25 ± 2.87
*P*-Value	0.808	0.164	0.029

#### 3.4.2 Hormone levels

We further evaluated changes in serum sex hormone levels, including E_2_, FSH, and LH, at 3, 6, and 12 months. Both treatment groups showed a similar trend of increased E_2_ levels and decreased FSH and LH levels (**Table 5**, **Supplementary Figure 1**). However, between-group comparisons indicated that these changes were not statistically significant at any time point (*P* > 0.05).

**Table 5:** Comparison of hormone level changes between two groups at 3, 6, and 12 months.

Index	Characteristics	3 Months	6 Months	12 Months
E_2_ (pg/mL)	Femoston	64.58 ± 76.41	37.03 ± 82.73	43.59 ± 78.43
	Femoston+HYKT	43.79 ± 67.50	39.57 ± 79.86	75.58 ± 106.17
	*P*-Value	0.168	0.361	0.335
FSH (mIU/mL)	Femoston	-47.51 ± 31.92	-39.49 ± 45.89	-40.68 ± 49.07
	Femoston+HYKT	-54.33 ± 36.87	-51.33 ± 38.65	-40.14 ± 38.40
	*P*-Value	0.466	0.537	1.000
LH (mIU/mL)	Femoston	-18.38 ± 18.53	-18.70 ± 18.37	-15.06 ± 19.57
	Femoston+HYKT	-19.17 ± 27.56	-20.77 ± 17.63	-19.40 ± 14.67
	*P*-Value	0.425	0.943	0.532

#### 3.4.3 Bone turnover markers

Changes in bone turnover markers (BTMs), specifically P1NP and β-CTX, were evaluated at 3, 6, and 12 months to assess bone formation and resorption following drug treatment. Over the course of treatment, levels of these markers showed an overall decreasing trend in both groups; however, between-group comparisons of changes from baseline at each follow-up time point revealed no statistically significant differences (**Table 6**).

**Table 6:** Comparison of changes in P1NP and β-CTX between two groups at 3, 6, and 12 months.

Index	Characteristics	3 Months	6 Months	12 Months
P1NP	Femoston	-5.48 ± 41.07	-23.84 ± 47.44	-36.81 ± 40.51
	Femoston+HYKT	17.22 ± 64.96	1.29 ± 49.27	-31.20 ± 58.50
	*P*	0.021	0.357	0.427
β-CTX	Femoston	-0.03 ± 0.11	0.02 ± 0.19	-0.11 ± 0.11
	Femoston+HYKT	0.04 ± 0.25	0 ± 0.21	-0.04 ± 0.26
	*P*	0.172	0.76	0.134

#### 3.4.4 Correlation analysis

**Figure 2:**
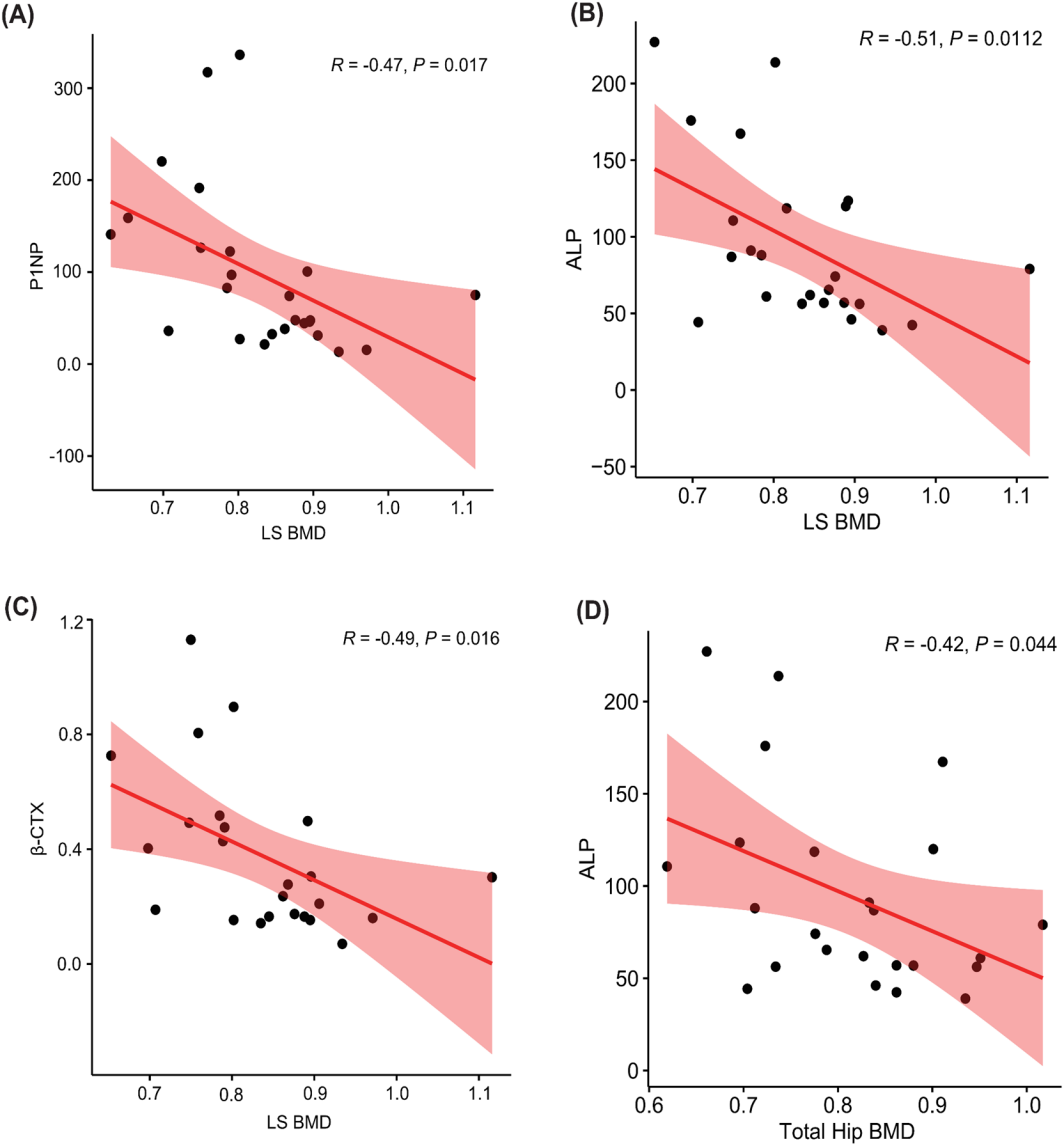
Correlation analysis between bone mineral density and bone turnover markers. **(A)** P1NP and lumbar spine BMD; **(B)** ALP and lumbar spine BMD; **(C)** β-CTX and lumbar spine BMD; **(D)** ALP and total hip BMD.

We then performed a correlation analysis to explore the relationships between BMD at different skeletal sites and various BTMs (**Table 7**, **Figure 2**).

**Table 7:** Correlation analysis between bone mineral density and bone turnover markers.

BTMs	Parameters	LS BMD	FN BMD	Left hip BMD
P1NP (ng/ml)	*r*	-0.475	-0.079	-0.198
	*P*	0.017	0.708	0.342
β-CTX (ng/ml)	*r*	-0.487	-0.263	-0.4
	*P*	0.016	0.215	0.053
ALP	*r*	-0.506	-0.249	-0.415
	*P*	0.012	0.242	0.044

Both P1NP and β-CTX showed significant negative correlations with LS BMD, suggesting that higher bone turnover is associated with lower bone density in the lumbar spine. However, no significant correlations were found with FN or total TH BMD. In addition, ALP demonstrated significant negative correlations with LS BMD and TH BMD, but not with FN BMD.

### 3.5 Safety

During the 12-month treatment and follow-up period, no cases of liver injury or renal dysfunction were observed in either group. Moreover, no fractures or other serious adverse events requiring hospitalization or treatment discontinuation were reported.

## 4 Discussion

POI is characterized by estrogen deficiency, which accelerates bone resorption and increases the risk of osteoporosis and fractures. Previous studies have reported significant BMD declines in women with POI compared with the general population, with reductions of 11.5% in the lumbar spine, 11.4% in the hip, and 9.1% in the forearm (14). However, high-quality clinical data on effective bone-protective interventions in POI remain limited (9, 15, 16). Therefore, this study aimed to evaluate whether combining KunTai capsules with estrogen therapy could enhance bone protection in women with POI.

HRT remains the cornerstone treatment for POI, alleviating hypoestrogenic symptoms and preventing osteoporosis and cardiovascular disease (17–19). However, evidence regarding the optimal HRT regimen remains limited, and clinical practice shows substantial variation in administration routes, dosages, and selection of estrogen and progestin formulations (20). Additionally, some patients are either contraindicated or unwilling/unable to receive HRT.

KunTai capsules, a traditional Chinese medicine approved by the National Medical Products Administration (NMPA) for ovarian insufficiency, consist of six components: *Rehmannia glutinosa*, *Coptis*, *Paeonia*, *Scutellaria*, *Equus asinus*, and *Poria cocos*. Previous studies have shown that KunTai capsules improve ovarian reserve function by increasing anti-Müllerian hormone (AMH) levels and antral follicle count (AFC) (21, 22). Notably, recent evidence indicates that AMH is not only an important biomarker of ovarian function but also stimulates human osteoblasts, activates osteogenic genes, upregulates transcription factors such as RUNX and OSX, and promotes mineralized nodule formation (23). Collectively, prior studies suggest that KunTai capsules may confer potential dual benefits, improving ovarian function while supporting bone health. However, these mechanisms were not directly assessed in the present study.

In patients with perimenopausal syndrome, KunTai capsules have been shown to significantly reduce the K-score and Menopause-Specific Quality of Life (MENQOL) score, thereby improving quality of life (24). One of the main components, Baicalin, has been found to enhance the viability of granulosa cells in aged mice, inhibit cell apoptosis, and increase estradiol and progesterone secretion (25). Furthermore, KunTai capsules have demonstrated significant clinical efficacy in patients with POI, effectively regulating E_2_, FSH, and LH levels, with both efficacy and safety superior to hormone therapy alone (26–28). The underlying mechanisms may involve protection of ovarian ultrastructure, reduction of follicular atresia and cell apoptosis, and an increase in the number of quiescent and growing follicle numbers (29, 30). Taken together, these studies provide preliminary evidence supporting the protective effect of KunTai capsules on ovarian function.

Preserving or increasing BMD and reducing fracture risk are primary objectives in maintaining bone health in POI patients (31). Previous studies have shown that in patients with POI (aged 18–44 years), lumbar spine bone mineral density increased significantly during two years of HRT, while total hip and femoral neck BMD remained stable throughout the treatment period (9). The spine is more sensitive to estrogen and responds to treatment more rapidly than the hip (32), which may explain the regional specificity of this improvement. In the present study, lumbar spine BMD increased significantly in both groups, with greater improvements at L2-L3 observed in the combination group. These findings indicate that the addition of KunTai capsules was associated with enhanced lumbar spine BMD compared with estrogen therapy alone. However, the underlying mechanisms remain unclear and require further investigation.

A baseline imbalance in lumbar spine BMD was observed, with the combination therapy group presenting a lower mean value compared with the Femoston-only group. To account for this difference, the primary analysis evaluated the change in BMD from baseline to 12 months (ΔBMD). This approach assesses treatment response relative to each participant’s baseline value. The greater increase in lumbar spine BMD observed in the Femoston+KunTai group supports a potential additional benefit of the combination therapy. Nevertheless, the baseline imbalance should be considered when interpreting the findings.

P1NP and β-CTX are sensitive biochemical markers of bone turnover, reliably reflecting bone metabolic activity. A previous study evaluated serum P1NP and β-CTX in healthy Chinese postmenopausal women, revealing that both markers first increased during the earlier postmenopausal period and then maintained at a high level in the following decades (33). Postmenopausal hormone replacement therapy (HRT) reduces the turnover rate of the mineralized bone matrix, thereby slowing bone loss. A previous study evaluated changes in bone turnover markers among postmenopausal women receiving HRT and found that serum P1NP levels decreased by 42% after 12 months of treatment (34). Another study assessed the effects of estrogen therapy on bone turnover markers in postmenopausal women. Compared with baseline values, serum β-CTX levels decreased significantly at 12 and 24 weeks after treatment, while P1NP levels showed a significant reduction after 24 weeks (35). To our knowledge, the present study is the first to evaluate the effects of KunTai capsules on bone turnover markers in patients with POI. Decreases in bone turnover markers were observed in both groups during treatment. However, no significant between-group differences were detected, and therefore no definitive conclusions can be drawn regarding the specific effects of KunTai capsules on bone turnover.

Our correlation analysis further demonstrated a negative association between BTMs and lumbar spine BMD, confirming that elevated bone turnover corresponds to lower bone mass in POI. Evaluating these markers alongside BMD measurements may improve clinical assessment of bone metabolism and help tailor therapeutic strategies to prevent osteoporosis in this high-risk population.

Elevated FSH has been identified as an important factor contributing to decreased BMD in perimenopausal women. Sun et al. demonstrated that FSH can directly bind to FSH receptors (FSHR) on the surface of osteoclasts in hypogonadal mice, thereby promoting bone resorption and reducing bone mass (36). Similarly, Lana et al. reported a positive correlation between serum FSH concentrations—rather than estradiol levels—and bone loss at the spine and femoral neck in women with primary POI (37). In the present study, both treatment groups exhibited increased serum E_2_ levels, along with decreases in FSH and LH levels from baseline, consistent with previous findings. Notably, the combination of KunTai capsules and hormone therapy led to a significantly greater reduction in FSH levels at 12 months compared to the control group, suggesting that the combination regimen may confer additional bone-protective effects through modulation of FSH levels.

In addition to hormone assessments, menopausal symptoms were evaluated at 3, 6, and 12 months of treatment using the modified K-score. The modified K-score, an updated version of the original scale, is widely employed in China to assess the severity of menopausal symptoms (38). Our results showed a significant reduction in the modified Kupperman score in the observation group, which was notably greater than that in the control group at 12 months. This suggests that the combination of KunTai capsules and Femoston provides enhanced relief for menopausal symptoms in POI patients. Importantly, the addition of KunTai capsules did not increase the incidence of adverse events.

The main limitation of this study is the relatively small sample size. In addition, the primary endpoint was analyzed using the Full Analysis Set based on available data. Lumbar spine BMD was assessed only at baseline and 12 months. The relatively high dropout rate (29.8%) at 12 months further limits the robustness of the findings. The study period overlapped with the COVID-19 pandemic, and restrictions on movement and hospital visits may have contributed to loss to follow-up, potentially introducing attrition bias. Furthermore, the absence of a placebo control group represents another limitation of this study and may have influenced subjective outcomes. Larger studies with extended follow-up are warranted to confirm these findings.

## 5 Conclusion

In conclusion, the addition of KunTai capsules to estrogen therapy was associated with greater improvement in lumbar spine bone mineral density at 12 months in women with POI. No significant between-group differences were observed in bone turnover markers or hormone levels. Further studies are needed to confirm these findings and clarify potential mechanisms.

## Data Availability

The raw data supporting the conclusions of this article will be made available by the authors, without undue reservation.

## Supplementary material

Supplementary Table 1 Comparison of Baseline Hormone Levels Between Two Groups Before and After 3, 6, and 12 Months of Treatment.
