# Supplementary material for: KunTai capsules combined with Femoston in premature ovarian insufficiency: a randomized controlled trial of the effects on bone mineral density
Source: Front Endocrinol (Lausanne). 2026 Mar 26;17:1746993. doi: 10.3389/fendo.2026.1746993 (PMC13061734; doi:10.3389/fendo.2026.1746993)
Supplement: Supplementary Table 1 — Comparison of Baseline Hormone Levels Between Two Groups Before and After 3, 6, and 12 Months of Treatment. [file Table1.docx]

**Supplementary Table 1.** **Comparison of Baseline Hormone Levels Between Two Groups Before and After 3, 6, and 12 Months of Treatment**

| Index | Characteristics | Baseline | 3 Months | 6 Months | 12 Months |
| --- | --- | --- | --- | --- | --- |
| FSH (mIU/mL) | Femoston | 33.56±38.31 | 92.93±76.07^*^ | 70.73±52.01^*^ | 71.41±71.07^*^ |
|  | Femoston+HYKT | 29.24±37.13 | 75.24±82.61^*^ | 64.16±75.23^*^ | 96.69±119.36^*^ |
| FSH (mIU/mL) | Femoston | 77.28±34.11 | 29.62±23.11^*^ | 37.73±24.67^*^ | 43.68±35.02 |
|  | Femoston+HYKT | 93.42±44.58 | 42.80±37.40^*^ | 43.59±36.90^*^ | 52.30±47.68^*^ |
| LH (mIU/mL) | Femoston | 32.67±15.94 | 14.38±12.17^*^ | 17.06±13.59^*^ | 19.73±17.58^*^ |
|  | Femoston+HYKT | 36.98±23.00 | 20.43±23.19^*^ | 19.98±17.89^*^ | 19.42±18.76^*^ |

* Within-group comparison vs. pre-treatment, P<0.05.
